# Physiological causes of transplantation shock on rice growth inhibition and delayed heading

**DOI:** 10.1038/s41598-021-96009-z

**Published:** 2021-08-19

**Authors:** HyeonSeok Lee, WoonHa Hwang, JaeHyeok Jeong, SeoYeong Yang, NamJin Jeong, ChungKuen Lee, MyoungGoo Choi

**Affiliations:** 1 Crop Production & Physiology Division, National Institute of Crop Science, Rural Development Administration, Wanju, 55365 South Korea; 2 Department of Crop Science and Biotechnology, Chonbuk National University, Jeonju, 54896 South Korea

**Keywords:** Plant sciences, Plant breeding, Plant development, Plant physiology

## Abstract

Transplanting is an important rice cultivation method; however, transplanting shock commonly affects grain yield, and the mechanisms underlying the inhibition of growth, development, and delayed heading caused by transplanting shock have not yet been clearly elucidated. Here, we investigated the effects of seedling age, temperature, and root damage during transplanting on growth, development, and time to heading, both under artificially controlled and natural day length. Additionally, we investigated the impact of seedling root growth space and the potential mitigating effects of residual seed nutrients on young transplanted seedlings. The delay in heading in transplanted versus directly seeded plants was affected more by growth inhibition during the seedling period than by root damage during transplanting. However, root damage had an effect on the inhibition of leaf and tiller development, and the ratio of leaves to tillers increased because tiller development was inhibited more by transplanting shock compared with leaf development. Based on these findings, we propose factors reflecting the delay in growth due to transplanting shock that should be included for more accurate rice phenology modeling and suggest advantageous seeding conditions and transplanting methods for improved rice cultivation and yield in response to climate change.

## Introduction

Rice transplanting is a cultivation method that has been used for as long as rice has been grown. Although transplanting requires more water than direct seeding, it prevents uneven emergence as well as cold and heat damage during the initial growth stage and facilitates easy weed management^1,2^. The initial growth of rice plants after transplanting is greatly affected by temperature and solar radiation. Recent more frequent occurrence of abnormally high (e.g., during temporary heat waves) or low temperatures has increased the risk of damage, resulting in inhibition of rice development and growth^3–5^. All stages of rice growth are affected by temperature; however, the critical temperature at which heat damage occurs varies among the stages, being high during the vegetative growth stage but relatively low during the meiotic, heading, and ripening stages. Consequently, the reproductive stages are at greater risk of heat damage^6,7^. Because average ambient temperatures are expected to continue rising due to global warming^8^, it would be advantageous to implement temporal shifts in rice cultivation to facilitate optimal yields of high-quality rice by avoiding the occurrence of high temperatures during the reproductive stages^4,9^. Therefore, it is necessary to determine suitable seeding and transplanting periods by predicting the heading date in relation to the increase in average temperature.

A number of models for predicting the heading date of rice under the prevailing environmental conditions in different growing regions have been developed. These models are based on data such as the number of growing days to heading in relation to temperature and photoperiod, which are major environmental factors affecting the heading of rice^10–13^. Most models predicting the heading of rice reflect growth inhibition after transplanting due to transplanting shock (TR shock)^14,15^. The period of growth inhibition in rice plants caused by TR shock is determined based on the increase in the number of days from transplanting to heading compared with that for plants grown from direct seeding^16^. However, clarity is needed regarding the mechanism of growth inhibition by TR shock and the factors that delay development, growth, and heading^17^.

In general, damage to the shoots or roots during transplanting is known to have a major effect^16,18^. The findings of a study that analyzed growth differences in relation to the degree of damage to the shoots and roots during transplanting showed that leaf development and the heading date did not differ between plants subjected to root cutting (to a length of approximately 3 cm), compared with plants transplanted without root cutting. However, inhibition of leaf development was reported when all roots were cut to a length of 0–1 cm^19,20^. Although root damage during transplanting is considered inevitable, only some plants have roots severely cut to less than 1 cm. In another study that compared the growth of plants transplanted without root or shoot damage to that of direct seeding plants, growth inhibition, observed as reduced tillering, was noted after transplanting. These findings suggested that root cutting is not the only factor contributing to a delay in leaf development and heading after transplanting^17^. Many studies have reported that inhibition of leaf and tiller growth and development and delayed heading occurs in transplanted rice when compared with direct seeding cultivation and that these differences vary according to seedling age^21,22^.

If the transplanting time is the same, the heading date may be delayed as the seedling growth is shorter^17,21,22^, although the heading date may differ according to the variety and the climatic conditions after transplanting. However, no studies have examined whether these effects are due to TR shock caused by root cutting or some other physiological cause. Thus, further studies are needed to evaluate the effects of seedling age, climatic conditions, and TR shock on inhibition of the growth and development of rice and delayed heading after transplanting compared with direct seeding cultivation.

Accordingly, in this study, we aimed to determine the factors that should be considered in order to increase the accuracy of models for predicting the heading date of rice, taking TR shock into account by identifying the physiological causes of the delay in growth after transplanting compared with direct seeding cultivation. Our second aim was to comprehensively discuss the rice seedling and transplanting conditions required for adapting rice cultivation to mitigate the effects of climate change.

## Materials and methods

### Ethics statement

The authors declare that all methods were performed in accordance with the relevant guidelines and regulations. The test varieties were provided by SeoYeong Yang of the Rice production and Physiology Division, National Institute of Crop science. We obtained permission from the National Institute of Crop Science to use these varieties (https://www.nics.go.kr/apo/breed.do?m=100000128&homepageSecod=nics).

### Experimental materials and design

Four experiments were conducted at the artificial weather facility of the National Institute of Crop Science in Jeonju, South Korea (35° 49ʹ 19ʺ N, 127° 8ʹ 56ʺ E). Use of this facility enabled artificial control of light intensity, temperature, and humidity. The following rice cultivars, representing ecotypes with different maturation times, were used in experiment 1: Odae (early-maturing, SR5204-39-8-2), Daebo (mid-maturing, YR2124706801), and Saenuri (mid- to late-maturing, HR14026-B-68-1-5). After confirming that there were no differences between varieties in experiment 1, only two varieties (Odae and Saenuri) were used to increase the number of treatments and indicators for growth investigation.

A composite slow-release fertilizer based on 9 kg/10 *a* nitrogen, 4.5 kg/10 *a* phosphate, and 5.7 kg/10 *a* potassium at an area ratio corresponding to three plants (planting distance: 30 × 14 cm) instead of the pot area was used in experiments 1, 2, and 3. In experiments 1 and 2, seedlings for transplanting were sown and grown in 406-cell seedling trays, whereas direct seeding seedlings were sown and grown in 1/5000 a Wagner pots, at a density of three plants per pot. A staggered sowing time was implemented to produce seedlings of different ages.

### Experiment 1: the impact of root damage, temperature, and seedling age on time to heading

In this experiment, we investigated the impact of rice transplanting on the number of growing days to heading in relation to the possible interactions among root damage, temperature, and seedling age. For all cultivars, 7-, 14-, and 20-day-old seedlings were transplanted on the same day into 1/5000 a Wagner pots, at a density of three plants per pot, to match the growing conditions of the direct seeding plants. Four root damage treatments were set up during transplanting: (1) direct seedling (no root damage), (2) transplanting without root damage, (3) root damage by cutting roots to a length of 1.5 cm, and (4) root damage by cutting roots to a length of 3 cm. In order to transplant plants with as little root damage as possible (treatment 2), the plants were removed from the seedling trays with the soil intact around the roots, which was then detached by gentle rinsing with water. During the growing period, three temperature treatments were applied to transplanted and directly seeded seedlings of all ages; the average temperature conditions were 22 ℃ (maximum 28 °C/minimum 18 °C), 25 ℃ (maximum 30 °C/minimum 20 °C), and 28℃ (maximum 33 °C/minimum 23 °C). The day length was set at 12.5 h. Both temperature and day length were artificially controlled. The number of growing days from transplanting to heading was recorded for all treatments.

### Experiment 2: the impact of natural day length and temperature on time to heading

**Figure 1:**
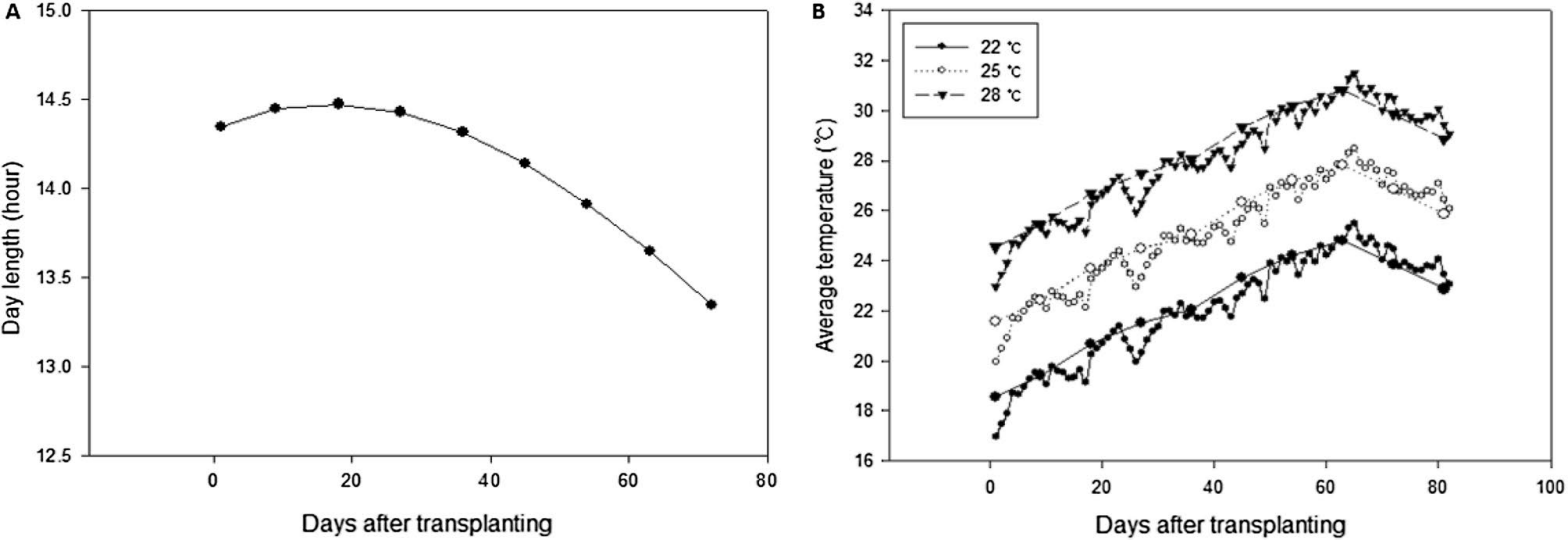
Changes in day length (**A**) and average temperature (**B**) during the growing period of rice from transplanting to heading.

Experiment 2 was conducted to confirm the heading delay results obtained in experiment 1 and to compare the number of growth days to heading between transplanted and directly seeded seedlings under the day length and temperature conditions occurring naturally during the rice growing season. For all cultivars, 10- and 20-day-old seedlings were transplanted on the same day into 1/5000 a Wagner pots, at a density of three plants per pot (to match the growing conditions of the directly seeded plants). At the time of transplanting, the same four root damage treatments applied in experiment 1 were set up following an identical protocol. The temperatures and day lengths from the time of transplanting to heading are shown in Fig. 1. The experimental seedlings were transplanted on May 29, and growth proceeded under the condition of increased day length until June 20 (23 days after transplanting), after which day length gradually decreased. Temperature during the growing period was artificially managed. The control group was grown at an average temperature of 25 °C (maximum 30 °C/minimum 20 °C). One treatment group was grown at a temperature 3 °C lower (i.e., at 22 °C), and a second treatment group was grown at a temperature 3 °C higher (i.e., at 28 °C). The temperature was averaged for 9 days after transplanting, and temperature conditions were changed every 9 days. The number of growing days from transplanting to heading and the growth and development of the transplanted and directly seeded seedlings were determined.

### Experiment 3: the effects of growth tray size on rice seedling growth and development

In experiment 3, we compared the growth and development of rice seedlings directly sown in 406-cell seedling trays (4 mL), nursery seedling trays (0.5 mL), and 1/5000 a Wagner pots (3958 mL; Fig. S1). In total, 15 rice plants were used repeatedly. The degree of leaf age development at 20 days after sowing was analyzed. The seedling trays were placed in a box containing soil to ensure a constant supply of fertilizer, and water supply was managed such that the soil surface was submerged to a depth of approximately 1 cm after emergence of the plants. During the growing period, the average temperature was 24 °C (maximum 29 °C/minimum 19 °C), and the day length was 12.5 h. Both temperature and day length were artificially controlled.

### Experiment 4: the effects of residual seed nutrients on growth and development of transplanted seedlings

Experiment 4 was conducted to further investigate the cause of the small degree of inhibition of growth, development, and time to heading noted in young seedlings that were transplanted. Specifically, we aimed to determine whether residual nutrients in the seeds (grains) after germination contributed to the observed changes. Four treatments were set up using 7-day-old seedlings: (1) seeds and roots maintained, (2) seeds maintained and roots cut, (3) seeds cut and roots maintained, and (4) seeds and roots cut. In total, 15 rice plants were used repeatedly. During the growing period, the average temperature was 24 °C (maximum 29 °C/minimum 19 °C), and the day length was 12.5 h. The number of growing days from transplanting to heading was determined.

### Dry weight, growth, and time to heading

Dry weight, growth, and time to heading were determined in the same way during all four experiments. For the analysis of dry weight, the shoots and roots of nine individuals were sampled and dried at 65 °C for 7 days. The shoots and roots were sampled separately. Three repetitions in batches of three individuals were sampled on days 1 and 17 after transplanting and used to examine changes occurring in the 16 days after transplanting. For growth analysis, the number of leaves and tillers was counted at intervals of 3–4 days from transplanting to heading. The heading date was analyzed as the number of days after transplanting and until the panicle emerged from the leaf sheath. Panicle emergence was examined at the same time each day (13:00 to 14:00).

### Root activity

Root activity was analyzed using the TTC reduction method^23,24^. In total, 12 rice plants in all treatments were sampled on days 1, 4, 7, 9, 11, 14, and 17 after root cutting and transplanting. Plants were sampled seven times on each day at 11:00 AM. After measuring the weight of the sampled roots, roots were immediately placed in a 15-mL tube containing 10 mL of 0.6% TTC solution and incubated at 30 °C for 24 h for drying. After washing with sterile water, 10 mL of 95% ethanol was added, and the mixture was boiled at 100 °C for 5 min to extract the dye. The extracted dyeing reagent was measured at 490 nm by adjusting the volume^23,24^.

### RNA extraction and gene expression

For RNA extraction, the second and third leaves of plants were sampled from each growth stage after transplanting and immediately frozen using liquid nitrogen. In total, 12 rice plants were used. Plant material for RNA extraction was sampled between 10:00 and 10:30 AM because Hd3a (a promoter of heading in rice) maintains a high expression level between 3 and 9 h after plants are exposed to light^25^. The samples were stored at − 80 °C. Total RNA was extracted according to the protocol described by Chang et al.^26^. cDNA synthesis was performed using Primescript RT reagent kit with gDNA eraser (Takara Bio Inc., Japan). For real-time polymerase chain reaction (PCR), SYBR Green (SYBR Realtime PCR Master Mix, Toyobo, Japan) was used as a fluorescent dye. The analysis was conducted using a Roter-Gene TM6000 (Corbett Research, Australia). Primer sequences are listed in Table S1.

### Statistical analysis

The following equation^27^ was used to model the development of leaf age and tillers based on the number of growing days from transplanting.

$$ F = \frac{{L_{\max } \;{\text{or}}\;T_{\max } }}{{1 + c^{{ - \left( {t - tm} \right) \times rF}} }}. $$

In this logistic model, *L*_*max*_ denotes the final number of leaves; *T*_*max*_ denotes the final number of tillers; *rF* is the rate of development until the final number of leaves or tillers; *t* denotes the number of days after transplanting; and *tm* denotes the time point at which half of the final number of leaves or tillers is reached, each referring to the time point when the rate of development reaches the maximum for the leaves or tillers. The values of *L*_*max*_, *T*_*max*_, *rF*, and *tm* are coefficients determined by nonlinear regression analysis, which was performed using Sigmaplot 11.1. The means were compared using Duncan’s test amd R program software (version 3.2.2); differences were considered significant when the *p* value was less than 0.05.

## Results

### Changes in growth and the number of growing days to heading in relation to seedling age, growth temperature, and extent of root damage

**Figure 2:**
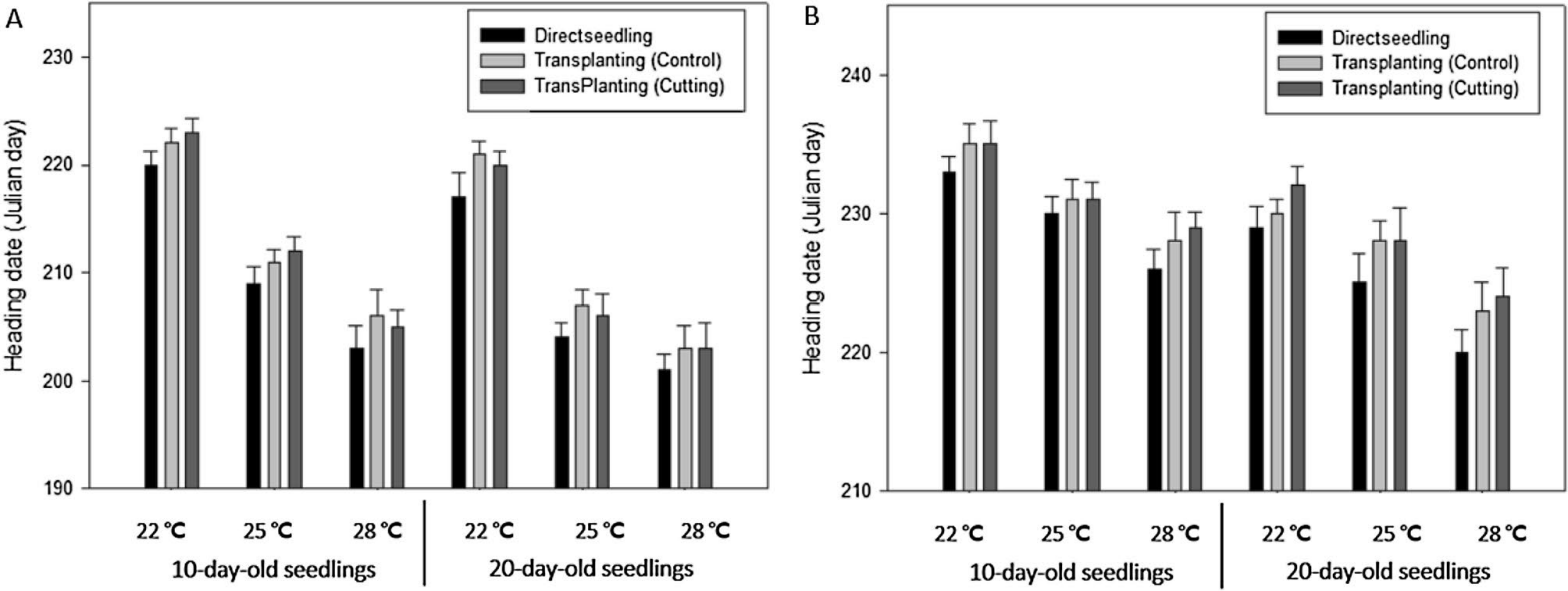
Differences in the time to heading of two rice cultivars in relation to root damage during transplantation, seedling age, and growth temperature. (**A**) Odae and (**B**) Saenuri. Error bars show standard deviations.

A comparison of the number of growing days to heading between directly seeded and transplanted plants showed that the direct seeding group reached the heading stage approximately 2–3 days sooner than the transplanted groups, regardless of average temperature, seedling age, and root damage during transplantation (Table 1). The difference in the number of growing days to heading between direct seeding and transplanting increased with seedling age by approximately 1.3, 2.4, and 2.7 days for the 7-, 14-, and 21-day-old seedlings, respectively (Fig. S2). Regarding the effects of temperature, the difference in time to heading between direct seeding and transplanting was greater at 22 °C (2.6 days) than at 25 °C (2.0 days) and at 28 °C (1.9 days; Fig. S2). Root damage during transplanting of seedlings appeared not to affect the time to heading as there were no significant differences among the two root cutting treatments and the undamaged roots treatment (Table 1). Similar to experiment 1, the results of experiment 2 revealed that the direct seeding group took approximately 2–3 days less to reach heading compared with the transplanting groups, and there were no differences in time to heading in response to root damage during transplanting (Fig. 2).

**Table 1:** Growth duration of rice from transplanting to heading in relation to root damage during transplanting, seedling age, and growth temperature.

Seedling age (days)	Average temperature (transplanting to heading)	Growth duration (days)											
		Odae (early-maturing type)				Daebo (mid-maturing type)				Saenuri (mid-/late-maturing type)			
		DS	TP	TP (1.5 cm)	TP (3 cm)	DS	TP	TP (1.5 cm)	TP (3 cm)	DS	TP	TP (1.5 cm)	TP (3 cm)
7	22 °C	68^a^	69^a^	69^a^	68^a^	51^a^	51^a^	54^b^	53^b^	51^a^	52^a^	54^b^	52^a^
	25 °C	52^a^	54^a^	53^a^	54^a^	40^a^	41^a^	42^a^	41^a^	40^a^	41^a^	40^a^	41^a^
	28 °C	47^a^	48^a^	49^a^	48^a^	38^a^	39^a^	40^a^	39^a^	39^a^	40^ab^	41^b^	41^b^
	Average	55	57	57	57	42	44	45	44	43	44	45	45
14	22 °C	62^a^	64^b^	64^b^	64^b^	48^a^	51^bc^	50^bc^	52^c^	48^ab^	52^b^	52^b^	51^b^
	25 °C	47^a^	49^b^	48^b^	49^ab^	37^a^	42^c^	39^b^	38^b^	37^a^	39^b^	40^c^	41^c^
	28 °C	43^a^	45^b^	44^ab^	45^b^	35^a^	36^a^	37^b^	37^b^	36^a^	39^b^	38^b^	39^b^
	Average	51	53	52	53	40	43	42	42	40	43	43	44
21	22 °C	58^a^	61^b^	63^c^	61^b^	45^a^	49^b^	48^b^	48^b^	44^a^	46^b^	49^c^	47^b^
	25 °C	44^a^	47^b^	46^b^	46^b^	36^a^	39^b^	37^ab^	38^b^	34^a^	36^b^	37^b^	36^b^
	28 °C	40^a^	42^b^	43^b^	42^b^	32^a^	35^b^	34^b^	34^b^	33^a^	36^b^	36^b^	34^a^
	Average	47	50	51	50	38	41	40	40	37	39	41	39
Average		51	53	53	53	40	43	42	42	40	42	43	42

*DS* direct seeding, *TP* transplanting (no root damage), *TP (1.5 cm)* roots cut to 1.5 cm, *TP (3 cm)* roots cut to 3 cm.

Significant differences were evaluated using Duncan’s multiple range test at the 5% level.

Within each column, values followed by different letters (a to c) are significantly different (P < 0.05).

### Changes in initial growth after transplanting

**Figure 3:**
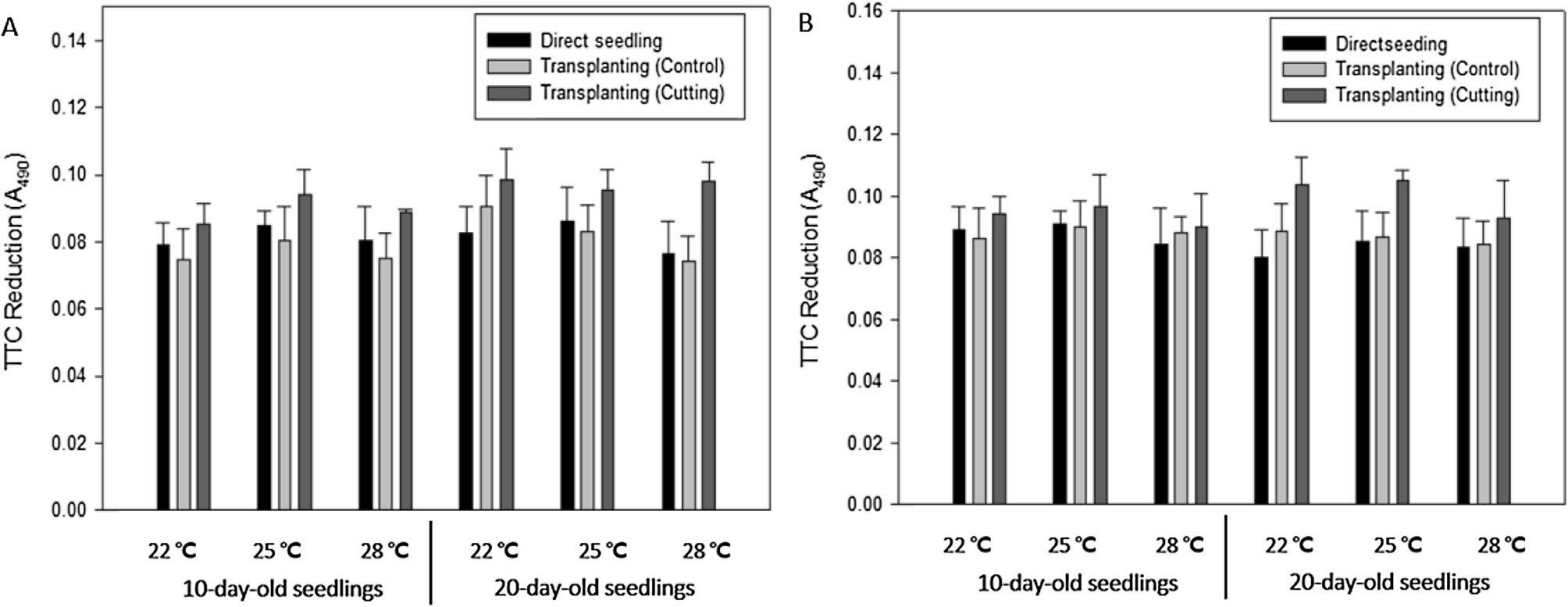
Difference in rice root activity (TTC reduction) in response to root cutting conditions, seedling age, and growth temperature. The analysis was conducted by sampling a total of seven times on days 1, 4, 7, 9, 11, 14, and 17 after transplanting and averaging the values for each day. (**A**) Odae, (**B**) Saenuri. Error bars are standard deviations.

To determine the possible cause of the delay in heading observed in transplanted compared with directly seeded plants, the root activity differences in response to root cutting were examined for 17 days after transplanting (Fig. 3). Root activity was higher in the group transplanted with root cutting than in the control transplanting group, regardless of average temperature and seedling age. This may be because the proportion of new roots was higher in the group subjected to root cutting. However, no significant differences in root activity among the different root cutting treatments were observed in relation to growth temperature and seedling age (Fig. 3).

**Figure 4:**
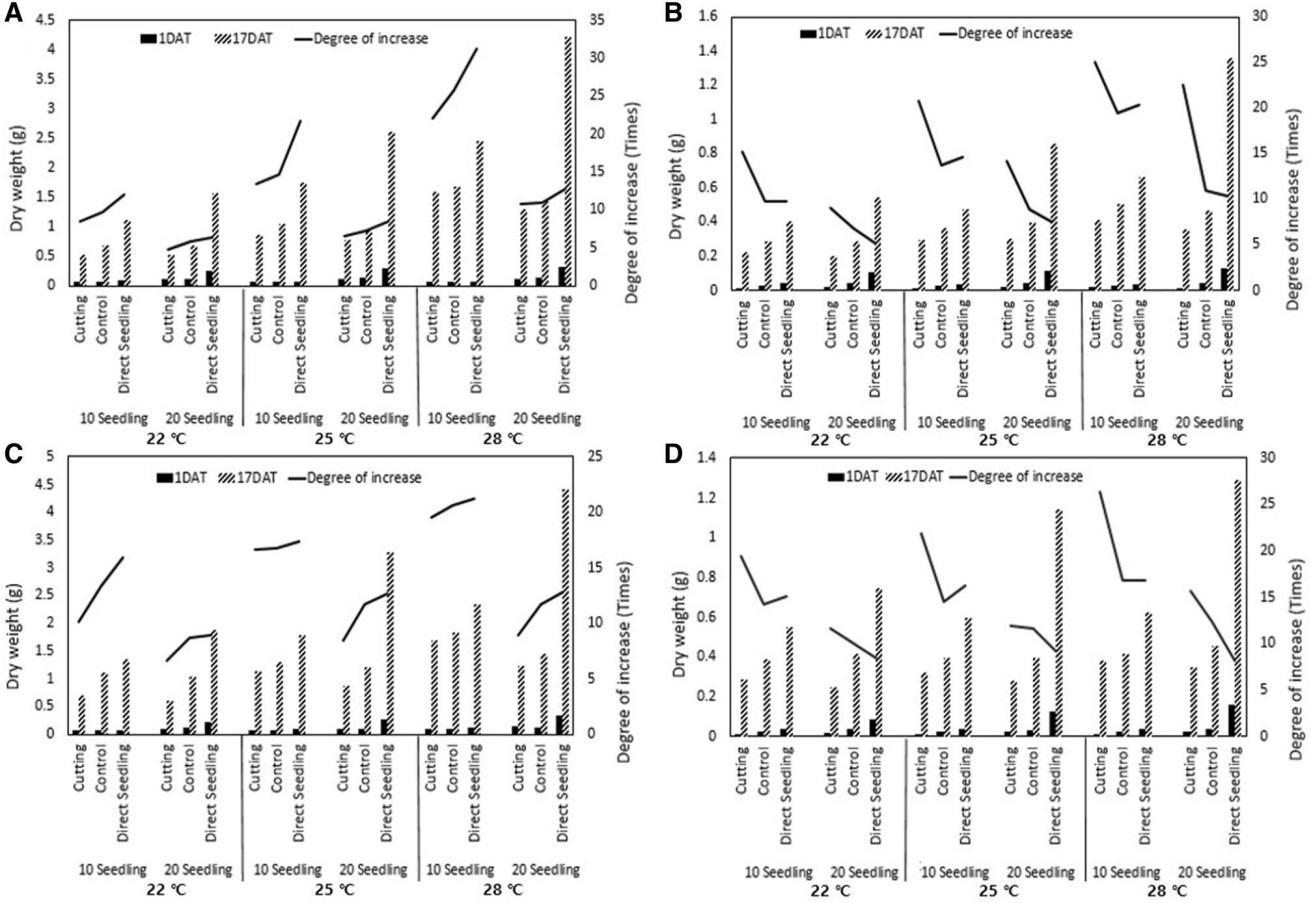
The dry weights of rice shoots and roots on days 1 and 17 after transplantation (left axis), and degree of dry weight increase (right axis). (**A**) Odae, shoot; (**B**) Odae, root; (**C**) Saenuri, shoot; (**D**) Saenuri, root.

To compare the extent of initial shoot and root growth among the treatments, shoot and root dry weights were determined immediately after transplanting and on day 17 of growth. Compared with the transplanting without root damage and transplanting with root cutting groups, the shoot and root dry weights of the direct seeding group were high, regardless of temperature conditions and seedling age (Fig. 4). The growth rate on day 17 compared with that on day 1 (after transplanting) was higher for the shoots, whereas the root growth rate was lower in the direct seeding group than that in the transplanting groups because root development was superior to that of the other groups, even on day 1 after transplanting (Fig. 4). With regard to root damage during transplanting, the root growth rate was higher in the root cutting group than in the group transplanted without root damage, whereas the shoot growth rate was lower (Fig. 4). The ratio of the shoots to roots was significantly higher for the root cutting group immediately after transplanting, following which it gradually decreased, reaching a ratio similar to that of the group without root damage and the direct seeding group by days 7–9 (Fig. S3). In the direct seeding group, although the growth rate on day 17 compared with that on day 1 (after transplanting) was higher in 20-day-old seedlings than in 10-day-old seedlings. However, the shoot and root dry weights were higher for the 20-day-old seedlings than the 10-day-old seedlings at 17 days after transplanting. In the transplanting group, as with the results of the direct seeding group, the growth rate on day 17 compared with that on day 1 (after transplanting) was higher in 20-day-old seedlings than in 10-day-old seedlings. However, the dry weights of the shoots and roots at 17 days after transplanting were higher in 10-day-old seedlings than in 20-day-old seedlings, unlike that in the direct seeding group (Fig. 4). A comparison of shoot and root dry weights on the basis of average temperature conditions showed that the influence of temperature on the growth rate of the shoots and roots was significantly greater for 10-day-old seedlings than for 20-day-old seedlings, and the temperature effect on shoot growth was particularly noticeable (Fig. 4, Supplemental Fig. S4).

### Development of leaves and tillers

**Figure 5:**
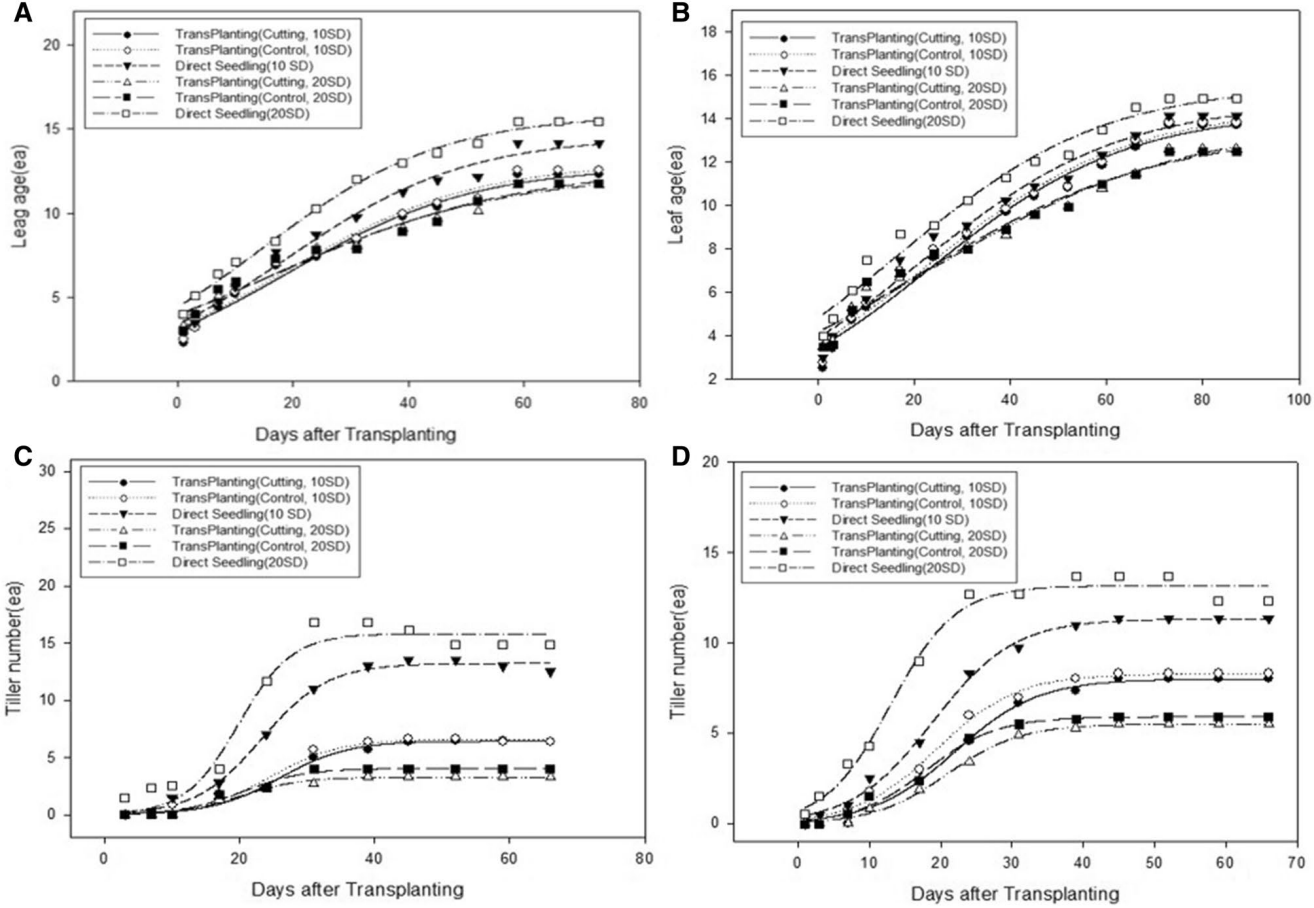
The degree of rice leaf age and tiller development after transplanting in response to root cutting conditions and seedling age. (**A**) Odae, leaf age; (**B**) Saenuri, leaf age; (**C**) Odae, tiller number; (**D**) Saenuri, tiller number.

**Figure 6:**
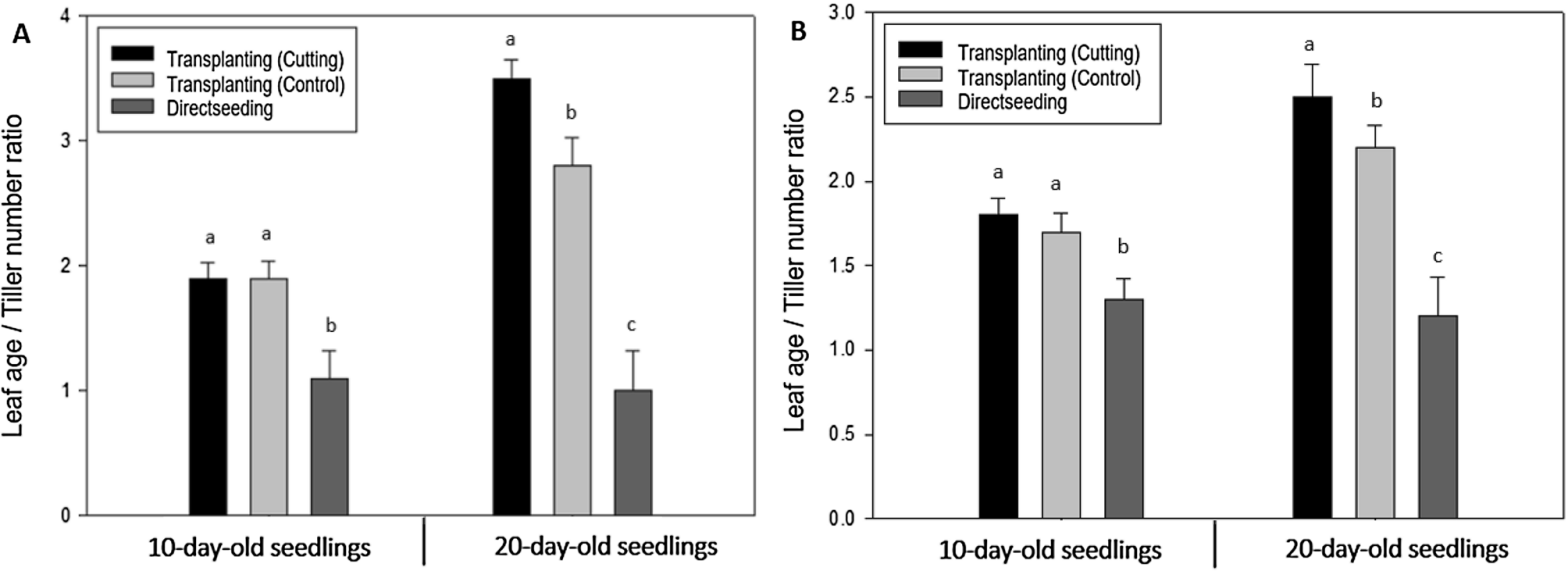
The difference in the ratio of the final number of leaves and tillers in response to root cutting conditions and seedling age in rice. (**A**) Odae, (**B**) Saenuri. Significant differences were evaluated using Duncan’s multiple range test at the 5% level. Error bars are standard deviations.

A logistic model was used to compare differences in the development of leaves and tillers among all treatments from transplanting to heading. The final number of leaves (*L*_*max*_) was greater in the direct seeding group than in the transplanting groups and was greater in 20-day-old seedlings than in 10-day-old seedlings in the direct seeding group (Fig. 5, Table 2). In the direct seeding group, the final number of leaves was smaller for 10-day-old seedlings than for 20-day-old seedlings, whereas in the transplanting group, the final number of leaves was smaller for 20-day-old seedlings than for 10-day-old seedlings. The rate of leaf development (*rF*) and the time point at which half of the rate of development of the final number of leaves was reached (*tm*) showed no difference for the 10-day-old seedlings in the direct seeding and transplanting (no root damage and root cutting) groups; however, the rate of leaf development (*rF*) was greatly reduced for the 20-day-old seedlings in the transplanting groups compared with the direct seeding group. The *tm* was greatly reduced in the direct seeding group compared with the transplanting groups. There were no differences in relation to the root cutting conditions (Fig. 5, Table 2). The final number of tillers (*T*_*max*_) was greater in the direct seeding group than in the transplanting groups. There was a greater difference in tiller development compared with leaf development between the direct seeding and transplanting groups. In relation to seedling age, the difference in tiller development between the direct seeding and transplanting groups was greater for the 20-day-old seedlings, which were seeded earlier, than for the 10-day-old seedlings in the direct seeding group (Fig. 5, Table 2). The ratios of the final number of leaves and tillers for each treatment are shown in Fig. 6. Compared with the direct seeding group, in the transplanting groups, tiller development appeared to be more inhibited compared with leaf development, resulting in a higher ratio of the leaf number to tiller number in response to transplanting. No differences between the transplanting without root damage and transplanting with root cutting groups were identified for 10-day-old seedlings, whereas the ratio of the leaf number to tiller number was slightly higher in the root cutting group for 20-day-old seedlings (Fig. 6). As shown in Table S2, inhibition of tiller development reduced the number of panicles, and the decrease in the number of panicles was similar to the results for tiller development. No differences between the transplanting without root damage and transplanting with root cutting groups were identified for 10-day-old seedlings, whereas the number of panicles was slightly lower in the root cutting group for 20-day-old seedlings. Similarly, the number of grains per panicle did not differ among treatments in 10-day-old seedlings, but decreased in transplanted seedlings compared with that in directly seeded seedlings in 20-day-old seedlings (Table S2).

**Table 2:** Parameters of the logistic function used to describe rice leaf age and tiller development by days after transplanting in relation to root cutting conditions and seedling age.

Rice types	Treatment		Development of leaf age				Development of tiller number			
			*L*_*max*_^a^	*r*_*F*_^b^	*t*_*m*_^c^	R^2^	*T*_*max*_	*r*_*F*_	*t*_*m*_	R^2^
Odae	10-day-old seed-lings	Transplanting (cutting)	12.79 (0.529)	0.061 (0.007)	18.82 (2.089)	0.97**	6.43 (0.131)	0.199 (0.022)	25.92 (0.652)	0.99**
		Transplanting (control)	13.02 (0.562)	0.061 (0.007)	18.51 (2.186)	0.97**	6.60 (0.233)	0.195 (0.036)	24.52 (1.154)	0.97**
		Direct seeding	14.56 (0.478)	0.064 (0.006)	18.73 (1.645)	0.98**	13.23 (0.211)	0.204 (0.018)	23.23 (0.521)	0.99**
	20-day-old seed-lings	Transplanting (cutting)	12.41 (0.660)	0.049 (0.019)	15.61 (2.828)	0.97**	3.29 (0.087)	0.216 (0.035)	20.22 (0.865)	0.98**
		Transplanting (control)	12.75 (0.979)	0.049 (0.015)	16.85 (3.198)	0.96**	4.03 (0.140)	0.222 (0.045)	20.41 (1.137)	0.97**
		Direct seeding	15.40 (0.238)	0.068 (0.006)	13.59 (0.958)	0.99**	15.83 (0.618)	0.253 (0.052)	19.82 (1.231)	0.95**
Saenuri	10-day-old seed-lings	Transplanting (cutting)	14.34 (0.513)	0.049 (0.005)	23.54 (2.171)	0.98**	8.00 (0.161)	0.172 (0.016)	22.04 (0.714)	0.99**
		Transplanting (control)	14.51 (0.542)	0.048 (0.005)	22.73 (2.276)	0.98**	8.28 (0.142)	0.177 (0.015)	19.48 (0.618)	0.99**
		Direct seeding	14.75 (0.558)	0.048 (0.005)	21.13 (2.296)	0.97**	11.32 (0.148)	0.176 (0.011)	18.93 (0.477)	0.99**
	20-day-old seed-lings	Transplanting (cutting)	13.85 (0.840)	0.037 (0.021)	22.28 (4.060)	0.97**	5.51 (0.081)	0.195 (0.015)	20.94 (0.504)	0.99**
		Transplanting (control)	13.84 (0.806)	0.039 (0.021)	20.05 (3.859)	0.96**	5.91 (0.104)	0.201 (0.019)	17.94 (0.611)	0.99**
		Direct seeding	15.57 (0.561)	0.064 (0.010)	16.92 (2.159)	0.97**	13.16 (0.247)	0.219 (0.024)	12.96 (0.636)	0.98**

^a^*L*_*max*_ is the final number of leaves, and *T*_*max*_ is the final number of tillers.

^b^*r*_*F*_ rate of development until the final number of leaves and tillers.

^c^*t*_*m*_* t* is the number of days after transplanting, *tm* is the time point at which half the number of final leaves and tillers is reached.

**p < 0.01.

## Discussion

### Physiological cause of growth inhibition and delayed heading due to TR shock

**Figure 7:**
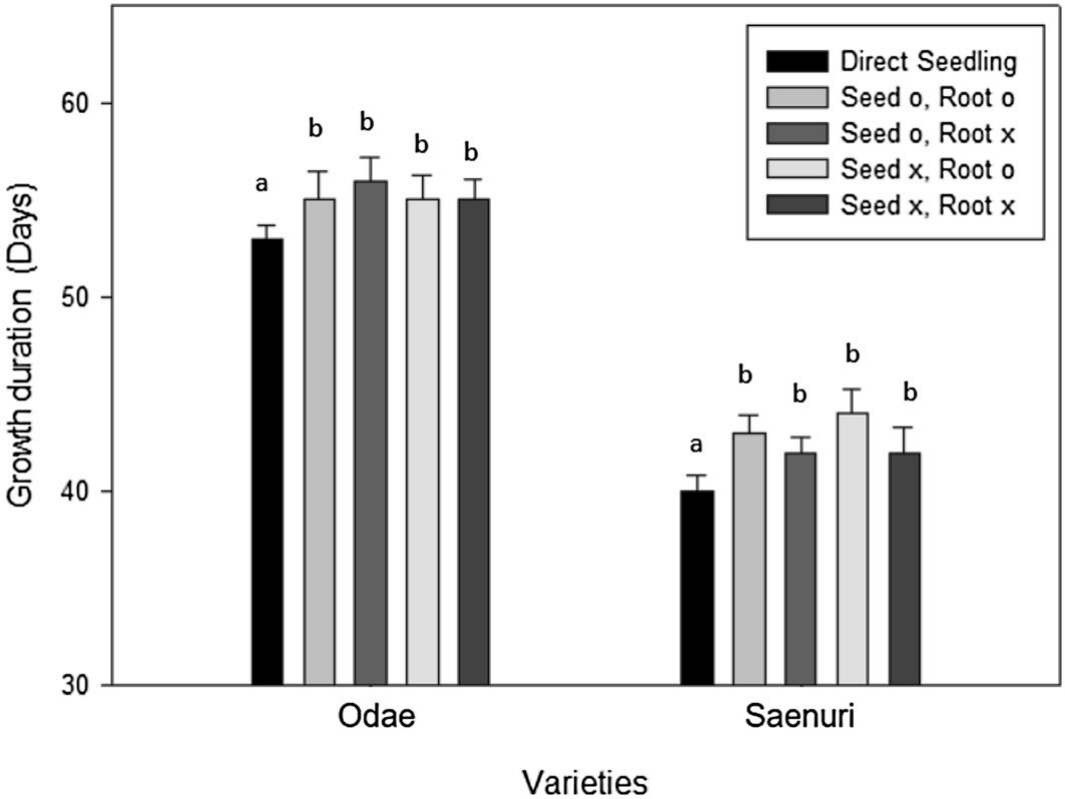
The growth duration under cutting (x) and noncutting (o) conditions for rice seeds and roots using 7-day-old seedlings. The roots were cut up to 1.5 cm in length. Significant differences were evaluated using Duncan’s multiple range test at the 5% level. Error bars are standard deviations.

The phenomenon of inhibition of growth and development after transplanting and delayed heading has been reported in previous studies. Several models predicting the heading date of rice also reflect the inhibition of growth due to TR shock after transplanting^10,14,15^. However, although a clear cause of such inhibition of growth has not yet been identified, damage to the shoots or roots during transplanting may be one cause^16–18^. In the current study, similar results obtained from repeat experiments showed that heading was delayed in the transplanting groups compared with that in the direct seeding group. By contrast, among the transplanting groups, there was no evidence that heading was delayed by root damage during transplanting. These results are in line with previous findings showing no differences in the heading date of plants with roots cut up to 3 cm in length and a difference in heading date within 0.5 days where roots were cut up to a length of 1 cm^20^. These results suggested that root cutting during transplanting is not the main factor that delays heading. By contrast, the development of leaves and tillers was greatly inhibited after transplanting compared with direct seeding. Given that tiller development was inhibited to a greater extent than leaf development, resulting in a higher ratio of leaf number to tiller number, tiller development was inhibited to maintain leaf development. Seedling age appeared to influence the differences in tiller and leaf development among the direct seeding and transplanting groups because the differences were greater in 20-day-old seedlings than in 10-day-old seedlings. Additionally, the older seedlings showed a difference in tiller development, even among the transplanting groups, depending on the root cutting conditions. The lower TR shock observed in younger seedlings may be related to nutrients remaining in the seeds (grains) after germination, which may facilitate early growth and development^28^. A comparison of the growth of 7-day-old seedlings after grain removal, in combination with root cutting, showed no differences in heading date among the transplanting treatments; however, a significant difference was noted among the transplanting and direct seeding groups (Fig. 7). Regarding tiller and panicle development, other than the difference between direct seeding and transplanting, development appeared to be inhibited when the grains or roots were damaged during transplanting, and this was evident for both cultivars (Table S3). Existing research shows that Hd3a, which promotes heading, also affects tiller development^29^; consequently, TR shock resulting from root cutting may first cause inhibition of tillering and then affect leaf development or cause delayed heading.

**Figure 8:**
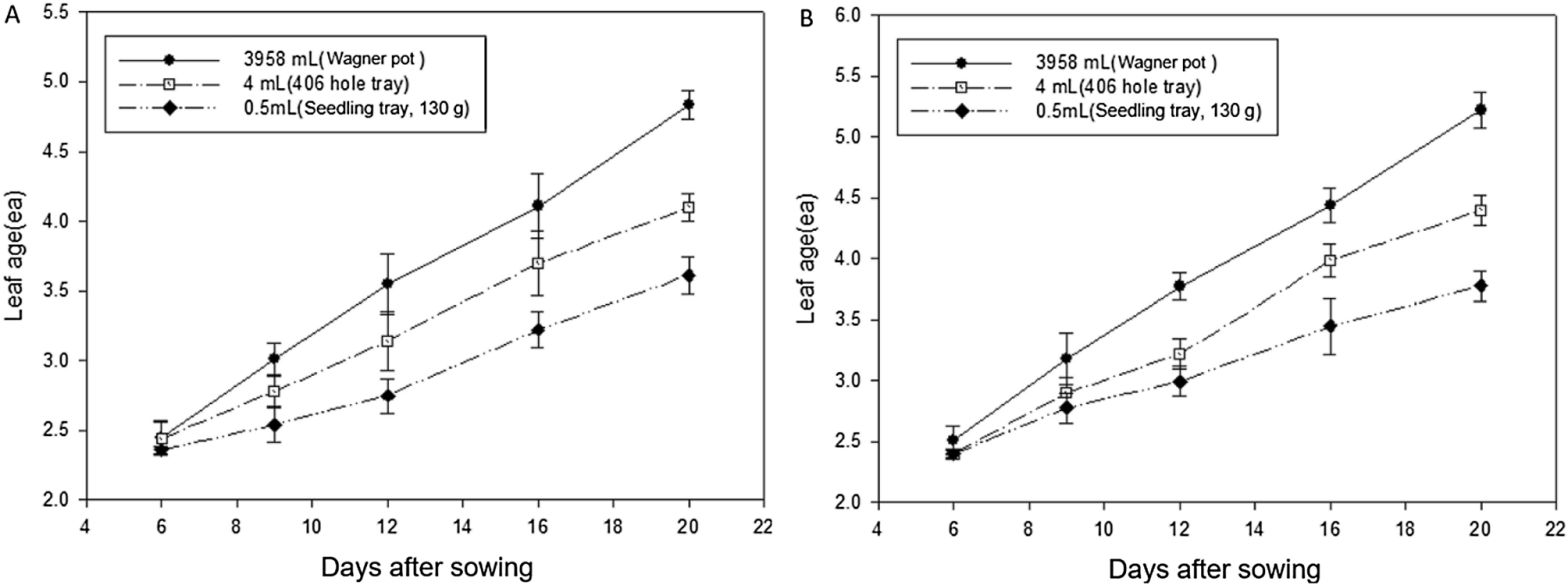
Changes in rice leaf development for 20 days after sowing in response to root growth space. (**A**) Odae, (**B**) Saenuri. Error bars are standard deviations.

After confirming that the effects of root cutting during transplanting were not the main factor delaying the heading date compared with direct seeding, an experiment was conducted to test the hypothesis that the delay in time to heading observed in the transplanting groups was related to inhibition of growth and development during the seedling stage (Fig. 8). The growth of seedlings is inhibited by competition between plants for light and nutrients, which becomes more intense as the seedling density increases^30^. In this study, containers of three different sizes were used for germination and early seedling growth (Fig. 8, Supplementary Fig. S1). Consequently, the possible impact of competition on inhibition of seedling growth and development was investigated by determining the effects of space available for root growth (as the treatment), rather than seeding density. For example, seedlings germinated and grown individually in each cell of the 406-cell seedling tray had a more limited space for root growth than the seedlings germinated and grown in Wagner pots. The growth and development of seedlings in each type of container was recorded for 20 days during the seedling stage. Differences in growth and development were evident from approximately day 6 to day 10, culminating in a difference of approximately 0.5–1 leaf between seedlings in the Wagner pots and those in the 406-cell seedling trays by day 20 (Fig. 8). Because one leaf develops every 3–5 days during the early stage of rice growth^31^, a delay in heading of approximately 2–3 days among the direct seeding and transplanting groups had already been initiated during the seedling stage. In the case of heading date, compared with the Wagner pots, there was a difference of 2–3 days in the 406-cell seedling trays and 5–6 days in the nursery seedling trays, consistent with the leaf age response (Fig. S5). This could explain why shorter seedling periods before transplanting resulted in smaller delays in heading caused by TR shock. In addition, a previous finding demonstrated that the growth rate of leaves did not show a significant difference after transplanting, regardless of the seedling period^32^, suggesting that inhibition occurred during the seedling period rather than being caused by TR shock after transplanting.

**Figure 9:**
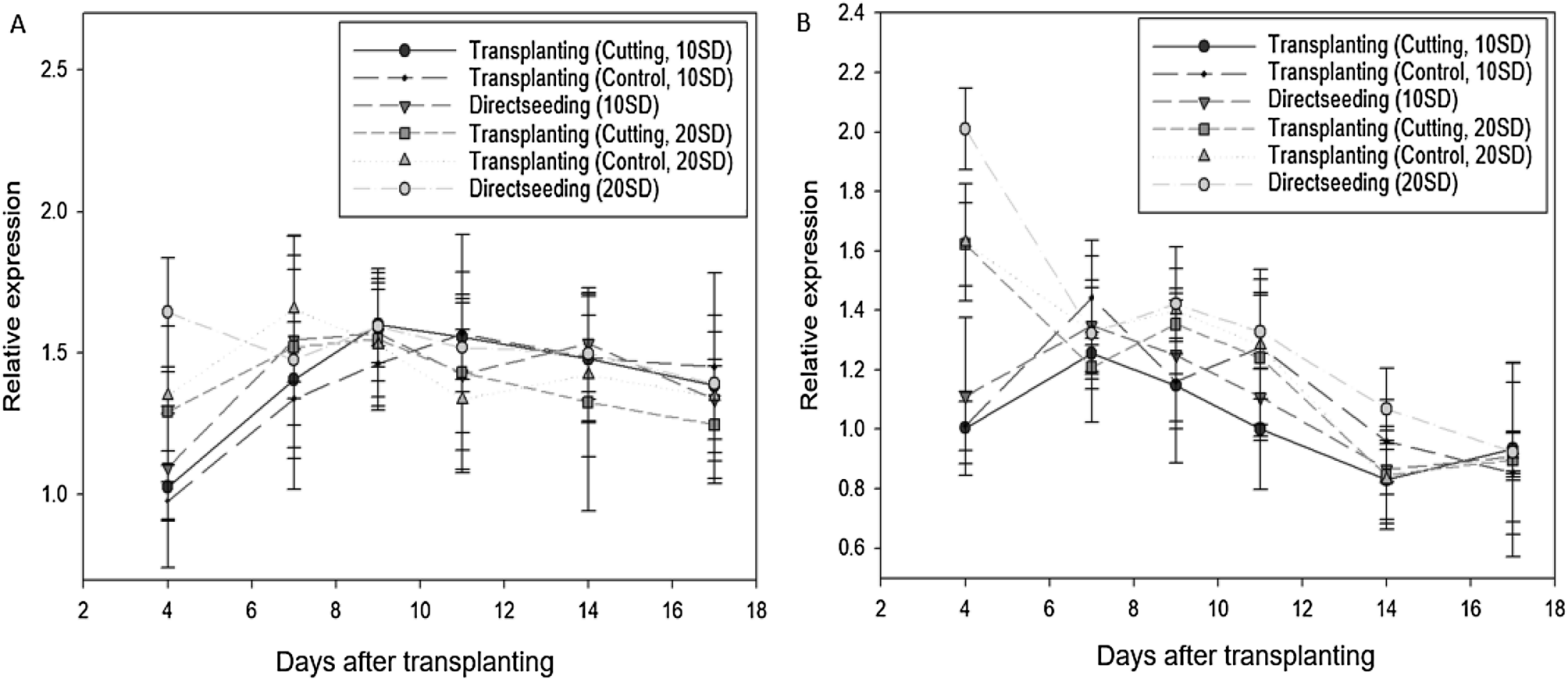
Changes in *Hd3a* gene expression after transplanting in response to root cutting conditions and seedling age. (**A**) Odae, (**B**) Saenuri. Error bars are standard deviations.

Rice begins photosensitization when the photoperiod becomes shorter than the critical photoperiod or when growth and development reaches a stage at which photosensitization can begin, after the vegetative growth stage^33,34^. In direct seeding, the growth space is larger, resulting in a faster rate of leaf development than that for individuals grown from transplanted seedlings. Consequently, directly seeded plants reached the photosensitization stage earlier. In the current study, the expression of Hd3a, which promoted the photosensitization reaction, was high from an early stage in individuals grown by direct seeding (Fig. 9), and the subsequent decrease in Hd3a expression may have been related to the increase in the photoperiod. Overall, the delay in leaf development and heading, together with impaired tillering in transplanted plants compared with those grown by direct seeding, appeared to be caused by growth inhibition during the seedling period, rather than through the effects of root cutting. This assertion was supported by the findings of a previous study, in which no differences in the period after transplanting to the first tillering were observed in relation to the number of seedling days, indicating that the difference in tillering was affected by the seedling period. However, Horie et al.^35^ reported different results.

In the main model used to predict rice heading date, the growth period is predicted by setting, as a parameter, the period during which growth stops and recovers as a consequence of TR shock after transplanting^10,14,15^. This model, reflecting TR shock, demonstrates a significantly improved prediction accuracy compared with the model that does not reflect TR shock^15^. Modeling using only the seedling period as a parameter can explain the difference in the recovery period after TR shock (R = 0.883), suggesting that the seedling period is a very important factor^15^. Testing the model using the seedling period, reproducible results were obtained under the experimental conditions in the current study. However, not only environmental conditions, such as temperature and solar radiation during the seedling period, but also conditions such as seedling space and seeding density change depending on the tester, which results in differences in leaf development at the time of transplanting and a reduction in predictability. Overall, the degree of leaf development at the time of transplanting is important for setting the recovery period due to TR shock after transplanting. Unless all seedling conditions can be made equivalent, the TR shock recovery period should be set using the degree of leaf age development at the time of transplanting as a parameter, rather than the seedling period.

The increase in average temperature due to global warming can greatly impact panicle infertility caused by heat waves during the heading and flowering stages of rice, decreasing the yield and quality of the harvested seeds because of high temperatures during ripening^36–38^. Given that the critical temperature is higher during the vegetative growth stage than the heading and ripening stages, seeding and transplanting should be postponed to avoid high temperatures during the reproductive stages^4,9^. Such temporal shifts in cultivation times to avoid high temperatures will result in photoperiod changes to short-day conditions during the vegetative growth period. Consequently, the vegetative growth period will be shortened, which will make it more difficult for rice plants to develop adequate leaves, tillers, and panicles. Under these environmental conditions, young seedlings that have relatively high temperature responsiveness, together with a relatively fast underground settlement and vegetative growth under high temperature conditions need to be used. For relatively young seedlings (10-day-old seedlings), the degree of inhibition is low during the seedling period. Despite the fact that the growth period is shortened under short-day and high-temperature conditions (Table 3), the period during which the leaves and tillers develop after transplantation increased, resulting in the production of more leaves and tillers compared with the older seedlings (20-day-old seedlings). These findings suggested that vegetative growth could proceed relatively well, even when rice plants were grown under short-day conditions at a high temperature if the time of seeding and transplanting of younger seedlings was delayed. Panicle length and the number of grains per panicle increase under short-day conditions^39–41^; however, tillering, which determines the number of panicles, is inhibited under short-day conditions. Consequently, the number of plantings at the transplanting site should be increased to compensate for reduced tillering and ensure that the required number of tillers is produced.

**Table 3:** The final number of rice leaves and tillers and environmental conditions during the growing period in relation to the number of seeding days and root cutting conditions.

Rice types	Treatment		Number of final leaves (ea)	Number of final tillers (ea)	Sowing to heading			
					Duration of growth (days)	Day length (h)	Average temperature (°C)	Accumulative temperature (°C)
Odae	10-day-old seed-lings	Transplanting (cutting)	12.3^c^	6.3^d^	73^c^	14.33	24.07	17.81
		Transplanting (control)	12.6^c^	6.5^c^	72^c^	14.33	24.02	17.54
		Direct seeding	14.2^b^	12.5^b^	70^d^	14.35	23.92	16.98
	20-day-old seed-lings	Transplanting (cutting)	11.7^d^	3.3^f^	77^a^	14.38	23.54	18.36
		Transplanting (control)	11.8^d^	4.1^e^	78^a^	14.38	23.59	18.63
		Direct seeding	15.1^a^	14.8^a^	75^b^	14.39	23.45	17.82
Saenuri	10-day-old seed-lings	Transplanting (cutting)	13.7^c^	8.0^c^	92^c^	14.16	24.71	22.98
		Transplanting (control)	13.8^c^	8.3^c^	92^c^	14.16	24.71	22.98
		Direct seedling	14.1^b^	11.3^b^	91^c^	14.17	24.69	22.72
	20-day-old seed-lings	Transplanting (cutting)	12.6^d^	5.3^e^	99^a^	14.22	24.38	24.38
		Transplanting (control)	12.5^d^	5.9^d^	99^a^	14.22	24.38	24.38
		Direct seeding	14.9^a^	12.3^a^	96^b^	14.25	24.31	23.58

Significant differences were evaluated using Duncan’s multiple range test at the 5% level.

Within each column, values followed by different letters (a to f) are significantly different (P < 0.05).

Many experiments have been conducted in rice cultivating regions around the world to identify the appropriate yield according to the seedling period^17,30^. However, the results for the appropriate seedling period have been interpreted differently for each experiment. In some experiments, only 2–3 years of yield and growth data are presented without a comprehensive summary of the cause analysis behind the selection of appropriate seedlings. It is not that the seedling period itself is important, but the criteria for setting appropriate seedling conditions vary depending on the geographic location of experimentation and the seedling environment. It is necessary to comprehensively analyze factors, such as the degree of leaf development at the time of transplanting after the seedling period, the environmental conditions (particularly temperature and photoperiod), and the characteristics of leaf and tiller development to establish suitable seedling and transplanting conditions in each region for adapting rice cultivation to climate change.

## Conclusion

In this study, we found that the delay in leaf development and heading of rice due to TR shock appeared to be affected more by inhibition of growth during the seedling period than by root cutting. However, root cutting increased the ratio of the final number of leaves to the final number of tillers by affecting tiller development. In the current model used for predicting the phenology of rice, the parameter is constructed by simply using the seedling period as a factor representing the period of growth inhibition due to TR shock. To increase the accuracy of prediction, it is necessary to include the number of leaves or the temperature and seedling conditions during the seedling period.

In response to global warming, for successful rice cultivation under short-day conditions at high temperatures, the period during which growth and development are inhibited in the seedling stage should be reduced, whereas the number of plantings should be increased to enhance the overall number of tillers produced and hence increase the panicle number. This will help to ensure sufficient growth and yield. Regarding the different flowering ecotypes, cultivars with a high rate of basic vegetative growth that can rapidly produce a sufficient number of tillers under short-day conditions should be used.

## Supplementary Information

Supplementary Information.

## Data Availability

All data and analyses needed to understand and evaluate the conclusions in the paper are presented in the paper or the Supplementary Materials. The source data for Tables 1, 2, 3, Figs. 1, 2, 3, 4, 5, 6, 7, 8, 9, Supplementary Tables S1–S3, and Supplementary Figs. S1–S5 are provided as Source Data files.
