# Supplementary material for: Physiological causes of transplantation shock on rice growth inhibition and delayed heading
Source: Sci Rep. 2021 Aug 19;11:16818. doi: 10.1038/s41598-021-96009-z (PMC8376942; doi:10.1038/s41598-021-96009-z)
Supplement: Supplementary file 1 — Supplementary Information. [file 41598_2021_96009_MOESM1_ESM.docx]

**Supplementary Materials**

**Table S1.** Gene-specific PCR primer sets for quantitative RT-PCR amplification.

| Enzyme | Gene  name | Accession  no. | Sequence | Amplicon  size |
| --- | --- | --- | --- | --- |
| Florigen | *Hd3a* | XM_015786465 | F: AGTTCCGGTCGGTCAGCAT  R: ACCGAGGTGGCAACAAAG | 117 |

**Table S2.** Differences in yield components in response to root cutting conditions and seedling age in rice.

| Rice types | Treatment | | Culm  length  (cm) | Panicle  length  (cm) | Number of  panicle  (ea) | Number of grains per panicle  (ea) | Ripening  ratio  (%) | 100-grain  weight  (g) |
| --- | --- | --- | --- | --- | --- | --- | --- | --- |
| Odae | 10-day-old  seed-  lings | Transplanting  (cutting) | 80.2^b^ | 17.8^ab^ | 6.3^c^ | 76.2 ^b^ | 79.2 ^a^ | 2.31 ^a^ |
|  |  | Transplanting  (control) | 81.0^b^ | 18.0^ab^ | 7.0^c^ | 76.5 ^b^ | 79.0 ^a^ | 2.25 ^a^ |
|  |  | Direct  seeding | 86.8^a^ | 17.4^b^ | 12.0^b^ | 75.5 ^b^ | 77.5 ^a^ | 2.29 ^a^ |
|  | 20-day-old  seed-  lings | Transplanting  (cutting) | 69.5^c^ | 16.5^c^ | 3.2^d^ | 70.2 ^d^ | 79.9 ^a^ | 2.23 ^a^ |
|  |  | Transplanting  (control) | 71.3^c^ | 17.3^b^ | 3.9^d^ | 73.1 ^c^ | 78.1 ^a^ | 2.19 ^a^ |
|  |  | Direct  seeding | 80.3^b^ | 18.5^a^ | 13.7^a^ | 78.4 ^a^ | 77.4 ^a^ | 2.24 ^a^ |
| Saenuri | 10-day-old  seed-  lings | Transplanting  (cutting) | 72.3^ab^ | 18.2^b^ | 6.7^c^ | 77.4 ^b^ | 85.4 ^b^ | 2.24 ^a^ |
|  |  | Transplanting  (control) | 72.3^ab^ | 18.5^b^ | 7.1^c^ | 77.1 ^b^ | 84.9 ^b^ | 2.21 ^a^ |
|  |  | Direct seedling | 73.5^a^ | 18.5^b^ | 8.2^b^ | 77.6 ^b^ | 86.6 ^ab^ | 2.26 ^a^ |
|  | 20-day-old  seed-  lings | Transplanting  (cutting) | 65.8^c^ | 17.9^b^ | 4.0^e^ | 76.9 ^b^ | 87.4 ^a^ | 2.18 ^a^ |
|  |  | Transplanting  (control) | 66.7^c^ | 18.2^b^ | 5.2^d^ | 76.8 ^b^ | 88.3^a^ | 2.28 ^a^ |
|  |  | Direct  seeding | 71.5^b^ | 19.5^a^ | 9.7^a^ | 79.4 ^a^ | 87.6 ^a^ | 2.34 ^a^ |

- Significant differences were evaluated using Duncan’s multiple range test at 5% level

**Table S3.** Rice growth and development under conditions of cutting (x) and noncutting (o) of seeds and roots using 7-day-old seedlings. The roots were cut to a length of 1.5 cm.

| Varieties | Treatment | Stem length  (cm) | Panicle length  (cm) | Number of tillers  (ea)_ | Number of panicles  (ea) |
| --- | --- | --- | --- | --- | --- |
| Odae | Direct seeding | 73.4^b^ | 20.4^a^ | 9.5^a^ | 7.6^a^ |
|  | Seed o, Root o | 78.2^a^ | 18.2b^b^ | 8.4^b^ | 7.4^a^ |
|  | Seed o, Root x | 78.5^a^ | 18.0^b^ | 6.6^bc^ | 5.0^b^ |
|  | Seed x, Root o | 77.1^a^ | 18.6^b^ | 6.0^c^ | 5.2^b^ |
|  | Seed x, Root x | 76.3^a^ | 18.5^b^ | 5.3^c^ | 4.0^b^ |
| Saenuri | Direct seeding | 71.4^a^ | 20.4^a^ | 9.6^a^ | 7.6^a^ |
|  | Seed o, Root o | 73.6^a^ | 19.4^a^ | 8.2^b^ | 7.0a^ab^ |
|  | Seed o, Root x | 73.4^a^ | 20.1^a^ | 7.6^b^ | 6.0^b^ |
|  | Seed x, Root o | 74.9^a^ | 19.8^a^ | 7.4^b^ | 5.5^b^ |
|  | Seed x, Root x | 72.5^a^ | 20.4^a^ | 7.2^b^ | 5.0^b^ |

Significant differences were evaluated using Duncan’s multiple range test at the 5% level

**Figures**


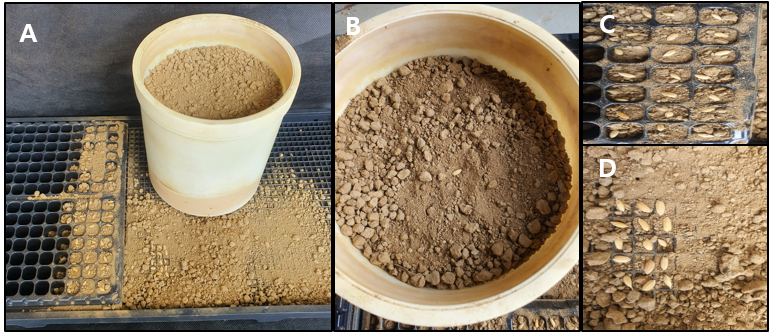


**Figure S1.** Photographs of Wagner pots and growing trays used to compare the growth and development of rice seedlings in experiment 3 (A). (B): 1/5000 a Wagner pots (3958 mL); (C): 406-cell seedling trays (4 mL); and (D): nursery seedling trays (0.5 mL).


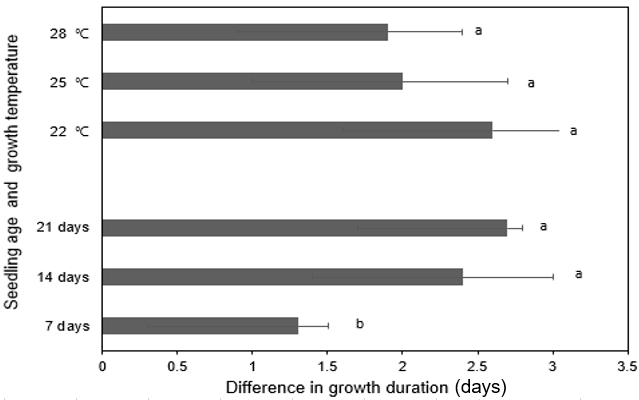
**Figure S2.** Differences in growth duration among direct seeding and transplanting treatments (average of undamaged roots, roots cut to 1.5 cm, and roots cut to 3 cm) in response to temperature and seedling age. Error bars are standard deviations. Significant differences were evaluated using Duncan’s multiple range test at the 5% level.


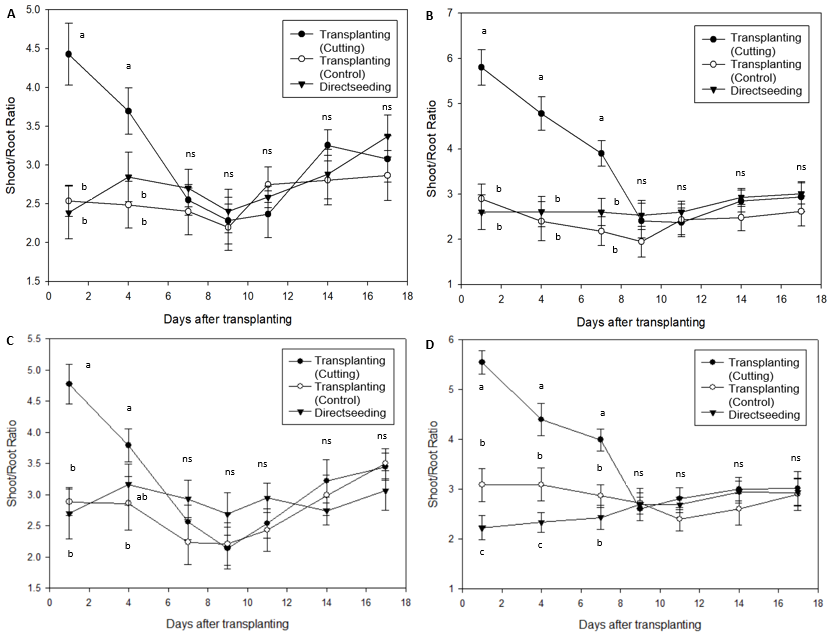


**Figure S3.** Changes in the ratio of shoots to roots after transplanting in response to root cutting conditions and seedling age. (A): Odae, 10-day-old seedlings; (B): Odae, 20-day-old seedlings; (C): Saenuri, 10-day-old seedlings; (D): Saenuri, 20-day-old seedlings. Error bars are standard deviations. Significant differences were evaluated using Duncan’s multiple range test at the 5% level.

**
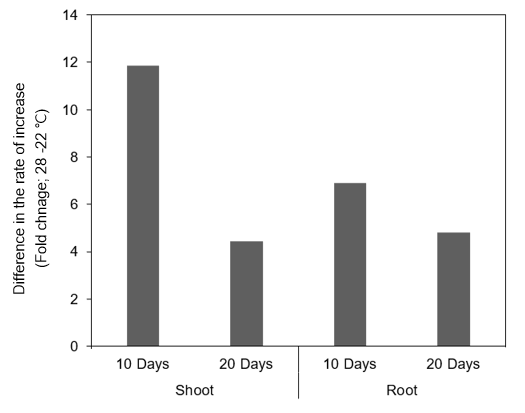
**

**Figure S4.** Difference in the rate of increase (fold change) in dry weight of shoots and roots for 17 days compared with 1 day after transplanting at temperature treatments of 28°C and 22°C for 10- and 20-day-old seedlings.

**
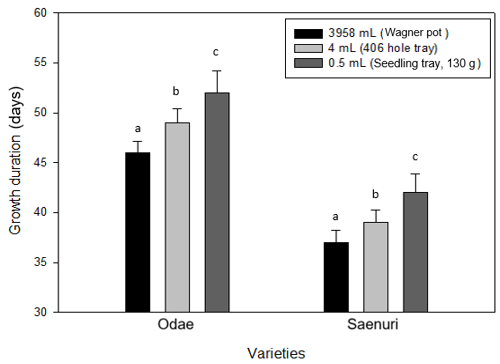
**

**Figure S5.** Changes in the growth duration from transplanting to heading according to seedlings with different leaf development in response to root growth space during the seedling period. Twenty-day-old seedlings were transplanted on the same day into 1/5000 а Wagner pots at a density of three plants per pot. Error bars are standard deviations.
